# A Review of Cyclic Imines in Shellfish: Worldwide Occurrence, Toxicity and Assessment of the Risk to Consumers

**DOI:** 10.3390/md22030129

**Published:** 2024-03-11

**Authors:** Sarah C. Finch, D. Tim Harwood, Michael J. Boundy, Andrew I. Selwood

**Affiliations:** 1 AgResearch, Ruakura Research Centre, Private Bag 3123, Hamilton 3240, New Zealand; 2 Cawthron Institute, Private Bag 2, Nelson 7042, New Zealand; tim.harwood@cawthron.org.nz (D.T.H.); michael.boundy@cawthron.org.nz (M.J.B.); andy.selwood@cawthron.org.nz (A.I.S.)

**Keywords:** risk assessment, pinnatoxins, gymnodimine, spirolides, shellfish toxins

## Abstract

Cyclic imines are a class of lipophilic shellfish toxins comprising gymnodimines, spirolides, pinnatoxins, portimines, pteriatoxins, prorocentrolides, spiro-prorocentrimine, symbiomines and kabirimine. They are structurally diverse, but all share an imine moiety as part of a bicyclic ring system. These compounds are produced by marine microalgal species and are characterized by the rapid death that they induce when injected into mice. Cyclic imines have been detected in a range of shellfish species collected from all over the world, which raises the question as to whether they present a food safety risk. The European Food Safety Authority (EFSA) considers them to be an emerging food safety issue, and in this review, the risk posed by these toxins to shellfish consumers is assessed by collating all available occurrence and toxicity data. Except for pinnatoxins, the risk posed to human health by the cyclic imines appears low, although this is based on only a limited dataset. For pinnatoxins, two different health-based guidance values have been proposed at which the concentration should not be exceeded in shellfish (268 and 23 µg PnTX/kg shellfish flesh), with the discrepancy caused by the application of different uncertainty factors. Pinnatoxins have been recorded globally in multiple shellfish species at concentrations of up to 54 times higher than the lower guidance figure. Despite this observation, pinnatoxins have not been associated with recorded human illness, so it appears that the lower guidance value may be conservative. However, there is insufficient data to generate a more robust guidance value, so additional occurrence data and toxicity information are needed.

## 1. Introduction

Some phytoplankton and benthic microalgae produce marine biotoxins which can accumulate in the flesh of filter-feeding shellfish species. These toxins can pose a health risk to humans, and illness due to their presence has been documented throughout history. Illnesses include paralytic shellfish poisoning (PSP) induced by the saxitoxin class of toxin, amnesic shellfish poisoning (ASP) caused by domoic acid, neurotoxic shellfish poisoning (NSP) caused by brevetoxins, diarrhetic shellfish poisoning (DSP) caused by okadaic acid and dinophysis toxins, and azaspiracid shellfish poisoning (AZP) caused by azaspiracids. The causative agents of these illnesses are well described, such that regulatory limits can be applied to protect the health of consumers and to facilitate trade [1,2]. These toxins have traditionally been detected and quantified using a mouse bioassay (MBA) [3]. The testing of some shellfish extracts on the NSP/DSP MBA, used for monitoring purposes, resulted in a potent response in mice with unusually short death times which was not consistent with NSP/DSP toxins. Analysis of the extracts showed no known toxins and further investigation resulted in the discovery of cyclic imines [4]. The quantification of shellfish toxins using the MBA is flawed, as it is based on the death time of mice which incorrectly assumes that the relationship between the death time and concentration is the same for each toxin type [5,6]. In addition, there are ethical concerns regarding the use of animals for routine monitoring of shellfish toxins [7], and for these reasons the MBA continues to be replaced by analytical chemical test methods. Development of LC-MS (liquid chromatography–mass spectrometry) methods now allows the detection of CIs alongside the ASP, DSP, NSP and AZP toxin groups [8].

## 2. Hazard Identification

**Figure 1:**
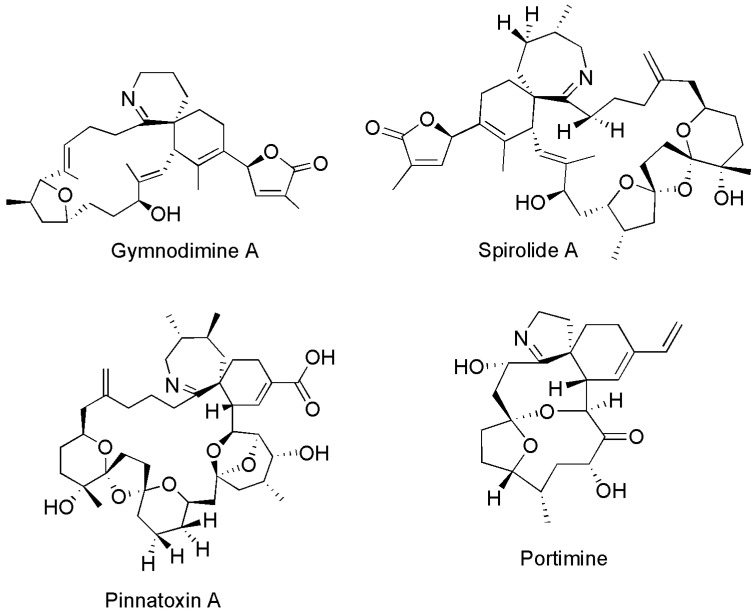
Chemical structures of Gymnodimine A, Spirolide A, Pinnatoxin A and Portimine.

The CI class of lipophilic shellfish toxins is comprised of gymnodimines (GYM), spirolides (SPX), pinnatoxins (PnTX), portimines, pteriatoxins, prorocentrolide, spiro-prorocentrimines, symbiomines and kabirimine (Figure 1). These compounds are structurally diverse but share a cyclic imine moiety (C-N double bond) as part of a bicyclic ring system. Extensive reviews of the chemistry and structures of CIs are available [9,10,11,12]. The mode of action of CIs is through the muscle and neuronal nicotinic acetylcholine receptors [9] and, through the use of cell and tissue assays, CIs have been shown to have high affinity and broad specificity for these receptor types [12,13]. CIs have been detected in multiple shellfish species from all around the world.

In 1980, a large-scale shellfish poisoning event in Japan occurred from the consumption of Taragi scallops. Initially, this was attributed to CIs, but contamination of the scallops with the pathogenic bacteria *Vibrio parahaemolyticus* was later found to be the cause [14]. Since then, despite the regular detection of CIs in shellfish, there have been no reported human intoxications. For this reason, CIs are currently not regulated, although the toxicology working group of the EU Community Reference Laboratory for Marine Biotoxins (CRLMB) have set a guidance level for SPXs [15], and an assessment by the French Agency for Food, Environment and Occupational Health and Safety (ANSES) has proposed a provisional acute reference dose for PnTXs [16].

CIs are considered by EFSA to be an emerging threat, and in this review, current knowledge on CIs will be summarized and the potential risk posed by this class of compound discussed. In addition, data gaps will be identified.

## 3. Hazard Characterization

The following section summarizes the discovery and toxicity of the various CIs.

### 3.1. Gymnodimines

In 1994, dredge oysters (*Tiostrea chilensis*) from Foveaux Strait (New Zealand) showed unusual toxicity in the routine MBA for lipophilic toxins. This resulted in the isolation and characterization of GYM A [4,17,18]. A later survey of historical New Zealand shellfish samples showed GYM contamination in eight species of shellfish from areas all around the New Zealand coast [19]. GYMs were found to be produced by the dinoflagellate *Gymnodinium* cf. *mikimotoi* (later reassigned as *Karenia selliformis*) and *Alexandrium peruvianum*. GYM B [20], C [21], D [22] and E [23] were isolated from these organisms along with 12-methyl GYM [24] and 16-desmethyl GYM D [23].

In addition to New Zealand, shellfish contaminated with GYMs have been detected in shellfish collected from Bosnia and Herzegovina [25], Croatia [25,26], France [27], Italy [28], Morocco [29], South Africa [30], Australia [31], China [32,33,34], Lebanon [35], Tunisia [36], Spain [37], New Caledonia [38] and Greece [39]. GYM A has been the dominant compound observed (Figure 1), but GYM B has been detected in shellfish from Lebanon and GYM D has been detected in shellfish originating from Spain. In addition, fatty acid esters of GYM A have been detected in shellfish from Tunisia [40] and China [41].

The characteristic feature of the toxicity induced by CIs is rapid mouse death. As enough pure GYMs became available, they were tested on mice. Mice dosed at lethal doses of GYM A were affected within 1 min, showing a rolling gait. Paralysis of the hind legs was then observed before respiratory distress and abdominal breathing. The respiratory rate of affected mice then became slower until death, typically 15 min post-dosing. Mice dosed with sub-lethal doses of GYM A showed paralysis and abnormal respiration but recovered within 30 min post-dosing. Pre-treatment of mice with the acetylcholine inhibitors physostigmine or neostigmine protected mice from the toxic effect of GYM A, which is consistent with their mode of action being on the nicotinic acetylcholine receptors [42]. This was confirmed in later work utilizing electrophysiological studies and binding assays [13]. Results of the available toxicity data for GYMs administered by intraperitoneal (i.p.) injection are presented in Table 1. The two LD_50_s reported for GYM A were consistent, but surprisingly the minimum lethal dose (MLD) and the “lethality” figures were considerably higher. However, these two results were presented purely as a value with no experiment details. It is therefore difficult to assess the validity of these figures. When tested in the same study, GYM B was shown to be considerably less toxic than GYM A by i.p. injection [13].

As expected, GYM A was less toxic when dosed orally compared to i.p. injection (Table 2). When dosed by gavage, symptoms of toxicity were the same as those observed with i.p. injection with death times of up to 12 min. This rapid death is unusual for orally administered compounds, suggesting that the method of dosing may be an issue. Unlike humans, the stomach contents of mice are paste-like, such that when a liquid is introduced it can flow around the solid mass to be rapidly absorbed by the duodenum [44]. This would result in an overestimation of toxicity, a phenomenon observed for many shellfish toxins [5]. In contrast, when administered with a solid matrix, the toxin will efficiently mix with the stomach contents of mice. Consistent with this hypothesis, mice dosed with GYM A in a solid matrix had longer death times and toxicity was greatly reduced compared to when the toxin was administered by gavage. Incorporation of GYM A consumed by mice resulted in no toxicity at a dose rate of 7500 µg/kg. Therefore, although highly toxic when injected by i.p., GYM A is of low toxicity when administered orally, making it unlikely to pose a food safety threat. Concentrations of GYMs in shellfish were regularly reported to be greater than 1 mg/kg shellfish flesh, but despite these very high concentrations there have been no reports of human illness [4].

### 3.2. Spirolides

Consistent with the discovery and isolation of GYMs, the SPXs were also identified due to the very rapid death of mice observed during routine monitoring of shellfish using the MBA. Shellfish collected from Nova Scotia, Canada in 1992 led to the isolation of SPXs B and D from the digestive glands of mussels and scallops [45]. In 1996, the same research group isolated SPXs E and F, again from Canadian shellfish [46], and in 2001, SPXs A and C as well as 13-desmethyl SPX C were isolated from the same source [47]. SPXs were found to be produced by the dinoflagellate *Alexandrium ostenfeldii* [48] and *A. peruvianum* [24] and further SPX analogues were isolated from these algal species. These analogues include 13-desmethyl SPX D [49], 20-methyl SPX G [50], 13,19-didesmethyl SPX C [51], SPX G [51], 27-hydroxy 13,19-didesmethyl SPX C [52], 27-hydroxy 13-desmethyl SPX C [52] 20-hydroxy 13,19-didesmethyl SPXs C and D [23] and SPXs H and I [53]. As observed for other CI classes, fatty acid esters of SPXs were detected [54]. SPXs were found in shellfish from Argentina [55], Croatia [26], France [39], Italy [28], Norway [56], Portugal [57], Slovenia [58], Spain [59], China [60], Lebanon [35], New Zealand [61], Greece [39] and the Netherlands [15]. The dominant SPX detected was 13-desmethyl SPX C, but SPX A, 13-desmethyl SPX D, 13,19-didesmethyl SPX C, SPX C, isoSPX C, 20-methyl SPX G, SPX D, 13,19-didesmethyl SPX C and iso13,19-desmethyl SPX C were also found.

The available information on the toxicity of SPXs administered to mice by i.p. injection is presented in Table 3. The symptoms of SPX toxicity were consistent with those observed for GYM, including the characteristic rapid death (3 to 20 min post-dosing). Any mice that survived for 20 min fully recovered. Some of the studies completed in different laboratories gave conflicting toxicities. For example, Munday et al. [62] reported the LD_50_s for 13-desmethyl SPX C and methyl SPX G to be 6.9 and 8.0 µg/kg, respectively, whereas Otero et al. [63] reported the LD_50_ of 13-desmethyl SPX C to be 27.9 µg/kg and the MLD of 20-methyl SPX G to be >63.5 µg/kg. The latter study is therefore reporting considerably less toxicity. However, comparison of results is not possible due to the lack of details regarding the mice used (strain, gender, state of alimentation) in the Otero et al. study [63]. This discrepancy could also have been due to the purity of the SPXs used. The study by Hu et al. [45] gave no experimental details, meaning that the validity of the data cannot be assessed. Based on the data presented in Table 3, 13-desmethyl SPX C, SPX C and 20-methyl SPX G are the most toxic, followed by SPX A, 13,19-didesmethyl SPX C, 27-hydroxy-13-desmethyl SPX C and 27-oxo-13,19-didesmethyl SPX C. SPXs E, F and H were of low toxicity to mice by i.p. injection.

There are less data available on the oral toxicity of SPX analogues, presumably due to the larger amount of compound required to perform the testing. The only study is that of Munday et al. [62], who tested SPXs using several different experimental protocols (Table 4). As expected, the oral toxicity of SPXs was less than that observed by i.p. injection and although clinical signs were the same as those previously observed, longer death times of up to 35 min were reported. SPX was more toxic to mice that had been fasted prior to dosing, thus highlighting the influence of mouse stomach contents. This phenomenon is seen regularly for shellfish toxins and the influence of stomach contents on toxicity has recently been thoroughly investigated [64]. Toxicity of SPXs was higher in mice that were dosed by gavage as opposed to those fed a matrix containing the toxin. This is not surprising, and as discussed in Section 3.1, the toxicity observed in mice voluntary fed is of greater relevance. None of the SPX analogues tested were associated with high toxicity when voluntarily fed to mice.

### 3.3. Pinnatoxins

The first pinnatoxin, PnTX A, was isolated in 1995 from the bivalve *Pinna muricata* originating from Okinawa, Japan [65]. PnTXs B, C and D were later isolated from the same Japanese bivalves [66,67]. In 2007, pacific oysters (*Crassostrea gigas*) from South Australia induced rapid deaths of mice in routine biotoxin monitoring using the MBA. This led to the isolation of PnTXs E, F and G [68]. At this point, the causative organism was unknown, but Rhodes et al. [69] determined it to be the dinoflagellate *Vulcanodinium rugosum* and PnTX H was isolated from this algal species [70]. As observed with other CIs, fatty acid esters were detected. In this case, it was fatty acid esters of PnTXs A and G [71]. PnTXs have been found to contaminate shellfish from Canada [71], Chile [72], Croatia [26], France [73], Ireland [58], Netherlands [58], New Caledonia [38] Norway [74], Spain [75], Mozambique [76], New Zealand [61], Slovenia [58], Italy [58] and Greece [39]. PnTX G is the most abundant analogue observed, but PnTXs A, D, E and F have also been detected in shellfish (Section 4). Although no human illness has been associated with PnTXs in shellfish, an outbreak of acute dermatitis in Cuba was linked to a bloom of *V. rugosum*. This affected 60 swimmers who required medical attention, but all fully recovered within 7–10 days [77]. Furthermore, artisanal fishermen suffered similar symptoms in the presence of *V. rugosum* [78]. It is clear that the *V.rugosum* bloom was the cause of the skin irritations and this bloom produced PnTX and portimine, but further work is required to determine whether these CIs are causal [77].

The acute toxicities of PnTXs are presented in Table 5 and Table 6. Early work in 1995 showed that natural PnTX A had an acute toxicity of 135–180 µg/kg by i.p. in contrast to the synthetic enantiomer which showed no effect even at 5000 µg/kg. The mixture of PnTXs B and C also showed high toxicity. However, as acknowledged in Table 5, these studies give no experimental details, so it is not possible to assess their validity. PnTx F was of higher toxicity than PnTXs E, G and H. PnTX D appears to be of lower toxicity than PnTX A (Table 5).

Only limited oral toxicity data are available for the PnTXs. By gavage, PnTX E had an LD_50_ of 2800 µg/kg. As discussed earlier, gavage gives an overestimation of oral toxicity and therefore the toxicity of PnTX E appears to be low. The acute toxicity of PnTXs F and G were 2.0 and 2.7 times lower by gavage than by voluntary feeding. Since voluntary feeding is the route of administration of most relevance to humans, these data are more informative than that determined by gavage. There was no difference between the toxicity of PnTX F to mice that had been fasted or fed prior to dosing. This is unusual and was not the case for SPXs or GYMs. The two independent determinations of the toxicity of PnTX G were reasonably consistent (150 and 208 µg, for the studies by Munday et al. [80] and Sosa et al. [81], respectively). The clinical signs of PnTX toxicity were the same as those observed for GYMs and SPXs. No observable adverse effect levels (NOAEL) for PnTXs F and G were determined to be 16 and 153 µg/kg, respectively [80]. It is interesting to compare the relative toxicities between the i.p. and oral routes of administration. GYM A is 78 times less toxic orally than by i.p. injection. Similarly, the relative toxicity of the SPXs were 32–73 times less toxic. In contrast, the oral toxicities of the PnTXs are only 3.2–9.4 times less than that determined by i.p. injection. Since toxicity via oral administration has the most relevance to human health, this difference is important. By i.p. injection, the SPXs are more toxic (6.9–37 µg/kg) than the PnTXs (14.9–400 µg/kg), but by oral administration PnTXs (50–400 µg/kg) are more toxic than the SPXs (500–1200 µg/kg). Based on these data, it appears that PnTXs are the sub-group of CIs that present the greater food safety risk.

### 3.4. Other Cyclic Imine Classes

Pteriatoxins (PtTX) A-C were isolated from the Okinawan bivalve *Pteria penguin* (Japan) and are thought to be derived from the metabolism of PnTX G in shellfish [68]. When tested on mice by i.p. injection, clinical signs were said to “resemble those of pinnatoxins” [82]. Acute toxicity data by i.p. injection are presented in Table 7. There are currently no oral toxicity data available.

A further class of CI, the prorocentrolides, were first isolated in 1988 from a culture of the dinoflagellate *Prorocentrum lima* collected from Okinawa, Japan [83]. A further prorocentrolide, prorocentrolide B, was later isolated from a *P. maculosum* culture and was described as inducing rapid death in mice by i.p. injection [84]. This rapid death is consistent with that observed for other CIs. A structurally related compound, spiro-prorocentrimine, was isolated from a *P. lima* strain collected from Taiwan [85]. All available toxicity data are presented in Table 7. As detailed in the table, a variable quantity of experimental details are published for each study. Prorocentrolide appears to be of moderate toxicity, although toxicity is merely described as “lethality”. In comparison, spiro-prorocentrimine was of low toxicity. Research into prorocentrolide analogues continues as they have been discovered to have in vitro antitumor activity and are therefore of interest as cancer therapeutic agents [86]. To date, eight analogues have been described in this sub-group of CIs. This includes the three detailed above, as well as prorocentrolide C, 4-hydroxy prorocentrolide, 9,51-dihydro prorocentrolide, 30-sulphate prorocentrolide and 14-0-acetyl-4-hydroxy prorocentrolide. These compounds were all isolated from *P. lima*, with prorocentrolide also being isolated from *P. caipirignum* [87].

**Table 7:** Toxicity of pteriatoxin, prorocentrolide and portimine analogues (µg/kg BW) to mice by i.p. injection.

Compound	Parameter	Acute Toxicity	Ref
Pteriatoxin A ^c^	LD_99_	100	[82]
Pteriatoxins B and C (1:1) ^c^	LD_99_	8	[82]
Prorocentrolide ^c^	“lethality”	400	[83]
Prorocentrolide B ^a^	Fast acting	ND	[84]
Spiro-prorocentrimine ^c^	LD_99_	2500	[85]
Portimine ^b^	LD_50_	1570 (1269–3080)	[88]

Figures in brackets indicate 95% confidence intervals; ^a^ mice were female CD-1; ^b^ details regarding the mice used were not available; ^c^ no experimental details were provided; ND = no data available.

Portimine, later named portimine A, was isolated from the benthic dinoflagellate *Vulcanodinium rugosum* collected from Northland, New Zealand [88]. Acute toxicity testing using mice showed it to be of low toxicity in comparison to other CIs (Table 7) [88] although it was highly toxic to mammalian cells in vitro. These in vitro effects include activity against cancer cells, which makes portimine an attractive target as a cancer therapeutic agent [89,90]. Portimine B was identified from *V. rugosum* isolated from Florida, USA. No in vivo toxicity data are available, but it also showed in vitro effects on mammalian cell lines, although with less potency than portimine A [91]. Portimine A has been detected in the digestive glands of shellfish collected from Ingril Lagoon, France, at concentrations of up to 69.3 µg/kg. These shellfish samples also contained GYMs (617.3 µg/kg), 13-desmethyl SPX C (25.9 µg/kg), PnTX A (6.6 µg/kg) and PnTx G (273.1 µg/kg) [92].

Another CI compound, kabirimine, was discovered from *V. rugosum* isolated from Okinawa, Japan. This compound, structurally related to portimine, was reported to have anti-respiratory syncytial virus activity [93]. No in vivo toxicity data are available [93].

Symbioimine and neosymbioimine were isolated from a symbiotic marine dinoflagellate, *Symbiodinium* sp., which is found in a wide variety of marine invertebrates [94]. These compounds showed in vitro activity, sparking interest in them as possible leads in the development of novel nonsteroid anti-inflammatory drugs [94].

## 4. Exposure Assessment

Worldwide occurrence data for CIs in multiple shellfish species have been collated (Table 8).

### 4.1. Gymnodimines

As described in Section 3.1, gymnodimines have been detected in a range of shellfish species from around the world (Table 8). However, compared to levels observed in other countries (max 103 µg/kg), flat oysters (*Tiostrea chilensis*) from New Zealand had remarkably high concentrations of GYMs (23,437 µg/kg).

### 4.2. Spirolides

The highest concentration of SPXs came from blue mussels (*Mytilus edulis*) collected from Norway in 2009, with 13 desmethyl SPX C observed at a level of 226 µg/kg shellfish flesh [56] (Table 8). SPXs have also been detected in processed shellfish samples. In 2020, 13-desmethyl SPX C was observed in the powder of mussels originating from New Zealand at concentrations of up to 98 µg/kg [110]. This level would be higher than what was observed in the original “wet” shellfish due to the removal of water during the powder drying process. Mussel powders are typically used as a dietary supplement, so only small amounts are consumed as a dose. In Portugal, “mussels in pickle sauce” were found to contain 66 µg/kg shellfish flesh of 13-desmethyl SPX C [58].

### 4.3. Pinnatoxins

The Ingril lagoon in France is a hot spot for PnTXs, with concentrations of up to 1244 µg total PnTXs per kg shellfish flesh being found in mussels, a concentration much higher than that seen in shellfish from anywhere else in the world [73]. PnTXs D, E and F have been found in oysters collected from the Rangaunu Harbour in New Zealand, with a total PnTX level of 198 µg/kg being reported. PnTX G was observed in mussels collected from Norway at a concentration of 115 µg/kg. Other than these examples, the concentration of PnTXs in shellfish was consistently <100 µg/kg irrespective of both the shellfish species and country of origin. PnTXs have also been detected in processed shellfish samples. Cooked mussels from Chile (5.2 µg/kg) [110] and frozen/canned mussels from Italy (4 µg/kg), Slovenia (3 µg/kg) and Spain (4 µg/kg) were all found to contain low levels of PnTX G. Furthermore, “mussels in brine” from Spain (6 µg/kg) and “mussels in tomato” from Slovenia (12 µg/kg) contained PnTX G [58].

## 5. Risk Characterization

To assess the risk posed by CIs, a safe concentration of each toxin in shellfish needs to be compared to the occurrence data presented in Table 8. Using animal toxicity data, an acute reference dose (ARfD) can by determined by taking the NOAEL and applying uncertainty factors (safety factors). The default uncertainty factor recommended and used by the EFSA is 100, which comprises a 10-fold uncertainty factor for inter-species variability and a 10-fold uncertainty factor for inter-human variability [111,112]. This is also the approach used by the UK government [113], but some organizations apply additional uncertainty factors due to limitations in the dataset. From the ARfD, the concentration in seafood that would not be exceeded by an average person (70 kg) eating a large portion size of shellfish (400 g) can be determined (safe concentration). The average bodyweight and large portion size are defined by EFSA [114], although the FAO/IOC/WHO (2004) Committee noted that a smaller portion size of 250 g would cover 97.5% of consumers [115].

The food safety risk posed by pteriatoxins, prorocentrolides, portimines, symbioimines and kabirimine can be regarded as low. Of these compounds, only portimine A has been detected in shellfish (69.3 µg/kg), and the acute toxicity of this compound is low (LD_50_ by i.p of 1570 µg/kg in mice). It should also be noted that this toxicity figure was generated by i.p. injection rather than by the more relevant oral route, meaning that toxicity was overestimated. Furthermore, the concentration given was µg/kg of digestive glands, where the toxin will be concentrated, rather than in whole shellfish flesh. These compounds therefore represent little risk to consumers and indeed they are under investigation as therapeutic agents. For the remaining CIs, concentrations considered safe in seafood were calculated using the above rationale (Table 9).

For GYM A, the highest dose tested on mice had no effect (7500 µg/kg), so although this figure was used in the calculation in Table 9, the actual “safe concentration” would be higher. This can be calculated when a true NOAEL is generated. Comparing the derived safe concentration of GYMs in shellfish (13,125 µg/kg) alongside the occurrence data shows that the amount of GYMs detected in most countries is only a fraction of this figure. The exception is the historic shellfish samples from Foveaux Strait, New Zealand, which contained GYM concentrations up to 23,437 µg/kg in shellfish flesh. However, in 1994, since oral toxicity was demonstrated to be low and no human illness had been detected, it was decided not to regulate GYMs despite the very high concentrations observed [4].

For SPXs, only oral LD_50_ data have been presented. An unpublished NOAEL of 320 µg/kg for 13-desmethyl SPX C can be used, yielding a guidance figure of 560 µg SPXs/kg shellfish flesh (Table 9). Previously, the toxicology working group of the EU Community Reference Laboratory for Marine Toxins (CRLMB) proposed a guidance level of 400 µg total SPXs/kg shellfish flesh [15]. However, the rationale for this limit was not mentioned, so it is hard to critique this objectively. There are no reports of SPXs exceeding the guidance figure in shellfish collected from anywhere in the world. The CRLMB specifies a figure for total SPXs and some shellfish samples have been shown to contain a mixture of SPX analogues. For example, Norwegian mussel samples contained 226 µg/kg 13-demethyl SPX C, 63 µg/kg SPX C, 49 µg/kg iso SPX C and 34 µg/kg 20-methyl SPX G. It is not valid to simply total the concentrations to estimate toxicity because the analogues have different toxicities. To be able to sum the concentrations of the different analogues, toxicity equivalence factors (TEFs) must be determined and applied. From the data presented to date, it is unlikely that SPXs pose any threat to human health.

PnTX G is the dominant PnTX species detected in shellfish and there are three studies that report an oral NOAEL or MLD (75–153 µg/kg). The lower figures of 75 and 120 µg/kg were generated by dosing PnTX G by gavage, whereas the higher figure of 153 µg/kg was obtained when mice were dosed with PnTX G by voluntary feeding. Since gavage is known to overestimate the toxicity of shellfish toxins, the figure determined by feeding is regarded as the most relevant, and using this figure yields a safe concentration of 268 µg/kg PnTXs in shellfish (Table 9). This figure is substantially higher than the 23 µg/kg proposed by Arnich et al. [95]. This study used the NOAEL determined by gavage (120 µg/kg) rather than that determined by feeding (153 µg/kg), but it was the application of additional uncertainty factors that was the major driver of the large discrepancy. In addition to the standard uncertainty factor of 100, Arnich et al. [95] also applied an extra uncertainty factor of 3 to account for insufficient data and a further uncertainty factor of 3 due to the severity and pattern of the dose–response curve, giving an overall safety factor of 900. Comparing our 268 µg PnTX/kg shellfish flesh value to the occurrence data reported in Table 8 shows that shellfish samples collected from France would at times exceed this figure. Using the 23 µg/kg value means that in addition to the samples from France, shellfish from Norway, New Zealand, Canada, Chile and Greece would also exceed this guidance figure on occasion. Given that shellfish samples from Ingril Lagoon in France have been reported to be contaminated with PnTX G at concentrations of up to 1244 µg/kg, which is 4.6 and 54 times the two proposed safe limits, it is perhaps surprising that no illness attributable to PnTX has been reported. It could be argued that illness is going undetected, but an investigation into PnTX contamination of shellfish collected from Rangaunu Harbour, New Zealand suggests that this may not be the case. Oysters collected from this harbour were analysed in 1993–2008 and found to contain PnTXs D, E and F at concentrations of 3.9, 126 and 68 µg/kg, respectively. Although these concentrations well exceed Arnich et al.’s guidance figure of 23 µg/kg for PnTXs, interviews conducted with 22 consumers of shellfish from this region in 2008, when the highest PnTX concentrations were detected, reported no adverse effects. These consumers reported their consumption of shellfish from this region to be 2.6 times/week for their entire lives. Consistent with the interviews, neither the local public health agency (Northland District Health Board, Te Runanga o Ngati Kahu) nor the national food safety regulatory agency (New Zealand Food Safety Authority) recorded any incidents of illness in Rangaunu Harbour residents over this time period [61].

Due to the lack of human illness from areas where PnTX concentrations in shellfish far exceed the safe level of 23 µg/kg, it can be concluded that this figure appears to be very conservative. In fact, the concentrations of PnTX in shellfish from some countries, such as Ingril Lagoon, France (1244 µg/kg), also exceed the higher guidance value of 268 µg/kg.

## 6. Discussion

In 2010, the EFSA produced a *Scientific Opinion on Marine Biotoxins in Shellfish-Cyclic Imines* [15]. In this report, the available knowledge was collated, and the risk posed by CIs discussed. The conclusion of this report was that “estimated exposure to SPXs does not raise concern for the health of the consumer”, although it was highlighted that this was based on limited data. The risk posed by the other CIs could also not be estimated due to lack of data. Since the 2010 *Scientific Opinion*, CI analysis methods have improved, more occurrence data have become available and additional toxicology has been performed.

However, despite this additional information, the data on CIs are still limited and the two proposed safe levels of PnTXs in shellfish (23 µg/kg by French researchers and 268 µg/kg by New Zealand researchers) should be considered provisional. To improve the accuracy and robustness of the CI risk assessment, additional data are required. This includes continuing to collect occurrence data in shellfish from around the world. New analysis methods for the CIs are also required, as many of these toxins exist as fatty acid esters which are not detected using current methods and their analysis poses a considerable technical challenge. In some cases, it has been found that these fatty acid esters can contribute >90% of the total CI concentration [36,71]. Current data illustrate that different analogues of the same CI sub-groups have different oral toxicities (e.g., PnTX G vs. PnTX F). Since the total concentration would therefore not be correlated to total toxicity, an adjustment must be made. This can be achieved by using TEFs, which compare the toxicity of each analogue to the parent compound of that toxin class. Appling TEFs allows a total concentration, in terms of parent toxin equivalents, to be calculated which contains the adjustment for toxicity differences. To be able to relate CI concentrations in shellfish to toxicity, TEFs must be determined and applied, which would require well characterized reference material to be isolated. To give greater certainty on the risk that CIs could pose to human health, better toxicity data need to be generated using pure compounds. In addition to further acute toxicity determinations, a sub-chronic study whereby the test compound is dosed for ≥21 days is required for the toxicological assessment of CIs. The results of a sub-chronic study would also allow a tolerable daily intake (TDI) to be generated. While these data gaps are relevant for each of the CI groups, it is the PnTXs that should be prioritised.

## 7. Conclusions

A review of all available data suggests that spirolides, portimines, pteriatoxins, prorocentrolide, spiro-prorocentrimine, symbiomines and kabirimine pose little risk to human health through consumption of shellfish. Despite occasional very high concentrations of GYMs and PnTXs in some shellfish collected from hot spots around the world, no illnesses have been reported, even when consumers have been directly questioned. Therefore, the risk of GYM and PnTXs to humans does not appear to be high. However, further occurrence and toxicity data are required to better define the risk posed by these compounds.

## Figures and Tables

**Table 1:** Toxicity of GYMs to mice (µg/kg BW) by intraperitoneal injection.

Compound	Mouse Strain	Gender	State of Alimentation	Parameter	Acute Toxicity	Ref
GYM A	?	?	?	“lethality”	450	[17]
GYM A	?	?	?	MLD	700	[43]
GYM A	Swiss albino	F	Fed	LD_50_	96 (79–118)	[42]
GYM A	Swiss Webster	M	?	LD_50_	80	[13]
GYM B	Swiss Webster	M	?	LD_50_	800	[13]

Figures in brackets indicate 95% confidence intervals; LD_50_ = median lethal dose; MLD = minimum lethal dose; ? = these experimental details are unknown.

**Table 2:** Oral LD_50_s of GYM A (µg/kg BW) using Swiss albino female mice.

Compound	Method of Administration	State of Alimentation	Acute Toxicity	Ref
GYM A	Gavage	Fed	750 (600–945)	[42]
GYM A	Over the tongue ^a^	?	4057 (3750–4390)	[42]
GYM A	Feeding ^b^	Fasted	>7500	[42]

Figures in brackets indicate 95% confidence intervals; ^a^ GYM A was mixed with ground mouse food to form a paste which was then administered over the tongue of the mouse; ^b^ GYM A was mixed with cream cheese which was eaten by mice within 30 s; ? = these experimental details are unknown.

**Table 3:** Toxicity of SPX analogues (µg/kg BW) administered to mice by i.p. injection.

Compound	Parameter	Acute Toxicity	Ref
Spirolide A ^a^	LD_50_	37 (35–44)	[62]
Spirolide B ^a^	LD_50_	99	[62]
Spirolide B ^c^	LD_100_	250	[45]
Dihydrospirolide B ^b^	MLD	>1000	[46]
Spirolide C ^a^	LD_50_	8.0 (4.6–16.0)	[62]
13-desmethyl spirolide C ^a^	LD_50_	6.9 (5.0–8.0)	[62]
13-desmethyl spirolide C	LD_50_	27.9	[63]
27-hydroxy-13-desmethyl spirolide C ^b^	MLD	>27	[52]
27-oxo-13,19-didesmethyl spirolide C ^b^	MLD	>35	[52]
13,19-didesmethyl spirolide C ^b^	LD_50_	32	[63]
13,19-didesmethyl spirolide C ^b^	MLD	30	[51]
Spirolide D ^c^	LD_100_	250	[45]
Spirolide E ^b^	MLD	>1000	[46]
Spirolide F ^b^	MLD	>1000	[46]
20-methyl spirolide G ^a^	LD_50_	8.0 (3.9–14.0)	[62]
20-methyl spirolide G	MLD	>63.5	[63]
Spirolide H ^a^	MLD	>2000	[53]

Figures in brackets indicate 95% confidence intervals. ^a^ Studies used fed, female, Swiss albino mice; ^b^ details of the mice used were not specified; ^c^ no experimental details were given.

**Table 4:** Oral toxicity of SPX analogues (µg/kg BW) to mice (from Munday et al. [62]).

Compound	Gavage		Voluntary Feeding	
	Fed	Fasted	Fed	Fasted
Spirolide A	550 (436–690)	240 (188–298)	1300 (1250–1580)	1200 (1047–3690)
Spirolide B	ND	440 (320–500)	ND	ND
Spirolide C	ND	53 (50–63)	780	500 (353–657)
13-desmethyl spirolide C	160 (123–198)	130 (87–166)	1000 (861–1290)	500 (381–707)
20-methyl spirolide G	160	88 (27–120)	ND	500 (381–707)

Figures in brackets indicate 95% confidence intervals; female, Swiss albino mice were used in all experiments; ND = no data available.

**Table 5:** Toxicity of PnTX analogues (µg/kg BW) in mice by i.p. injection.

Compound	Parameter	Fed Acute Toxicity	Fasted Acute Toxicity	Acute Toxicity	Ref
(+)-Pinnatoxin A ^b^	LD_99_	ND	ND	180	[65]
(+)-Pinnatoxin A ^b^	LD_99_	ND	ND	135	[79]
(−)-Pinnatoxin A ^b^	MLD	ND	ND	>5000	[79]
Pinnatoxin B/C 1:1 ^b^	LD_99_	ND	ND	22	[66]
Pinnatoxin D ^b^	LD_99_	ND	ND	400	[67]
Pinnatoxin E ^a^	LD_50_	45 (32–58)	ND	ND	[68]
Pinnatoxin E ^a^	LD_50_	57 (39.7–75.3)	48.0 (33.5–63.5)	ND	[80]
Pinnatoxin F ^a^	LD_50_	16 (12–23)	ND	ND	[68]
Pinnatoxin F ^a^	LD_50_	12.7 (9.5–14.6)	14.9 (12.6–15.8)	ND	[80]
Pinnatoxin G ^a^	LD_50_	50 (35–66)	ND	ND	[68]
Pinnatoxin G ^a^	LD_50_	48.0 (36.3–68.1)	42.7 (40.0–50.0)	ND	[80]
Pinnatoxin H ^a^	LD_50_	67 (63–79)	ND	ND	[70]

Figures in brackets indicate 95% confidence intervals; ^a^ Swiss albino, female mice were used; ^b^ no experimental details were provided; ND = no data available.

**Table 6:** Toxicity of PnTX analogues (µg/kg BW) to mice by oral administration.

Compound	Gavage		Voluntary Feeding		Ref
	Fed	Fasted	Fed	Fasted	
Pinnatoxin E	2800 (2380–3000)	ND	ND	ND	[80]
Pinnatoxin F	25.0 (19.1–35.1)	29.9 (25.0–32.0)	50.0 (39.4–62.8)	77	[80]
Pinnatoxin G	150 (105–199)	ND	400 (380–470)	ND	[80]
Pinnatoxin G	ND	208 (155–281)	ND	ND	[81]
Pinnatoxin H	163 (139–175)	ND	ND	ND	[70]

Figures in brackets indicate 95% confidence intervals; all used female Swiss albino mice, apart from the study by Sosa et al. [81] which used female CD-1s; ND = no data available.

**Table 8:** The maximum concentrations of GYMs, SPXs or PnTXs (µg/kg) detected in the whole flesh (unless otherwise specified) of different species of shellfish from around the world.

Country	Area	Year	GYM ^a^	SPX ^b^	PnTX ^c^	Ref
**Mussels (various species)**						
Argentina	Beagle Channel	2005–2007	<LOD	68 (1)	NT	[55]
Bosnia and Herzegovina	Bay of Neum	2017	11.4	NT	NT	[25]
Canada	Eastern Canada	2010–2011	NT	NT	83 (G), 1.5 (A)	[71]
Chile	Beagle Channel	2021–2022	NT	NT	100	[72]
Croatia	Istrian Peninsula	2018–2019	17.2	17.0 (1)	6.9	[26]
	Makarska City Bay	2017	7.4	NT	NT	[25]
France	Corsica	2021	3.5	Low	Low	[39]
	South Brittany	2005	<LOD	14 (1), 7 (2), 2 (3)	NT	[27]
	Atlantic Coast	2005	<LOD	68 (2), 19 (1)	NT	[27]
	Ingril Lagoon	2018	<LOD	12 (total)	473	[39]
	Ingril Lagoon	2010	NT	NT	1244	[73]
	Survey data	2013	NT	NT	89	[95]
	Ingril Lagoon	2021–2022	NT	NT	129	[96]
	Prevost Lagoon	2021–2022	NT	NT	129	[96]
	Thau Lagoon	2021–2022	NT	NT	58	[96]
	Vic Lagoon	2021–2022	NT	NT	318	[96]
Greece	Thermaikos Gulf	2008–2009	NT	26 (1)	NT	[97]
Ireland	Dublin	2015	<LOD	<LOD	4.6	[58]
Italy		2014–2015	12.1	29.2 (1) + (4)	<LOD	[28]
	Emilia Romagna Coast	2003	NT	13 (1)	NT	[98]
Mexico	Todos Santos Bay		NT	1.05 (1)	NT	[99]
Morocco	Essaouira	2014–2015	5.6	<LOD	NT	[29]
Netherlands	Ijmuiden	2015	<LOD	<LOD	5.1	[58]
New Zealand	Survey	1993–1999	2773	NT	NT	[19]
Norway		2009	NT	226 (1), 63 (5), 49 (6) 34 (7)	<LOD	[56]
	Hvaler	2009	NT	3 (5), 5 (7)	<LOD	[56]
	Nordreisa	2009	NT	52 (1), 34 (7)	7	[56]
	Nærøy	2009	NT	226 (1), 5 (3), 6 (6), 37 (7)	20	[56]
	Vadsø	2009	NT	42 (1), 63 (3), 16 (7)	10	[56]
	Vestvågøy	2009	NT	2 (1), 5 (7)	115	[56]
	Skjer	2002–2003	NT	44 (7), 103 total	NT	[50]
Portugal	Atlantic Coast	2009–2010	NT	2.2 (1)	NT	[100]
Slovenia	Marobor	2015	<LOD	33 (1)	<LOD	[58]
South Africa	Lambert’s Bay	2008	0.15	NT	NT	[30]
Spain	Galicia		NT	78 (1)	NT	[101]
	Galicia	2015	<LOD	6.9 (1)	3.1	[102]
	Catalonia	2012	NT	16 (1)	59	[75]
	Fangar Bay	2015–2021	Trace	Low	38	[103]
	Sant Carles de la Rapita	2018	<LOD	28 (1)	4	[58]
**Oysters (various species)**						
Australia	SE Queensland	2003–2005	43	NT	NT	[31]
China	Beibu Gulf	2017–2018	40.9	<LOD	NT	[32]
	Beibu Gulf	2018–2022	10.1	19.6 (1)	<LOD	[33]
	Daya Bay	2013–2014	2.64	NT	NT	[34]
Croatia	Istrian Peninsula	2018–2019	40.2	38 (1)	3.59	[26]
France	Atlantic Coast	2005	<LOD	47 (1)	NT	[27]
Lebanon	Tripoli, Beirut, Tyre		102.9 (B)	15.1 (1)	<LOD	[35]
Mozabique	Ihaca Island	2020	NT	NT	1.6	[76]
New Zealand	Foveaux Strait	1996	23,437	NT	NT	[19]
	Rangaunu Harbour	2008	<LOD	4.7 (1)	3.9 (D), 126 (E) 68 (F)	[61]
Spain	Galicia	2022	10 (DMD)	NT	NT	[104]
	Galicia	2021–2022	<LOD	21 (1)	NT	[105]
	Catalonia	2012	NT	6.6	<LOD	[75]
Slovenia	Marobor	2014–2015	<LOD	27	4	[58]
South Africa	Lambert’s Bay	2008	0.65	NT	NT	[30]
**Clams (various species)**						
China	Weihai, Shandong	2020	3.77	<LOD	NT	[106]
	Ganyu Harbour	2014–2015	5.96	<LOD	NT	[107]
Croatia	Cetina Estuary	2009–2010	6.14	2.1	NT	[108]
France	Ingril Lagoon	2010	NT	NT	95	[73]
	Brittany	2005	NT	8 (1)	NT	[27]
Italy	Goro, Caleri and La Spezia	2014	<LOD	<LOD	4	[58]
Lebanon	Tripoli, Beirut, Tyre	2019–2020	15.8 (B)	5.9 (1)	NT	[35]
New Caledonia	Noumea	2021–2022	22.6	<LOD	22.6	[38]
	Chukchi Sea	2014	<LOD	0.78 (1)	<LOD	[57]
	Lisbon	2015	<LOD	63 (1)	<LOD	[58]
Spain	Galicia	2005	NT	13 (1)	NT	[109]
Tunisia	Boughrara Lagoon	2000–2007	1290 (DG)	NT	NT	[36]
Mozambique	Inhaca Island	2020	NT	NT	4.5	[76]
**Cockles (*Acanthocardia turberculata* or *Cerastoderma edule*)**						
Croatia	Cetina Estuary	2009–2010	15.8	5.9 (1)	NT	[108]
Portugal	Lisbon	2015	<LOD	57 (1)	<LOD	[58]
Spain	Galicia	2022	8.8 (D)	NT	NT	[104]
**Scallops (*Aequipecten opercularis* or *Pecten novaezelandiae*)**						
Croatia	Istrian Peninsula	2018–2019	3.66	<LOD	<LOD	[26]
New Zealand	Survey	1993–1999	66.2	NT	NT	[19]
**Pipi (*Donax deltoides* or *Paphies australis*)**						
New Zealand	Survey	1993–1999	17.7	NT	NT	[19]
Australia	SE Queensland	2003–2005	220	NT	NT	[31]
**Abalone (*Haliotis iris*)**						
New Zealand	Survey	1993–1999	81.7	NT	NT	[19]
**Limpet (*Patella rustica* complex or *Patella intermedia*)**						
Lebanon	Tripoli, Beirut, Tyre	2019–2020	26.9 (B)	<LOD	<LOD	[35]
Portugal	Atlantic Coast	2009–2010	NT	1.9 (1)	NT	[100]
**Whelk (*Nucella lapillus* or *Neptunea varicifera*)**						
Portugal	Atlantic Coast	2009–2010	NT	1.1 (1)	NT	[100]
	Chukchi Sea	2014	<LOD	20 (1), 7.6 (5), 2.2 (8), 3.3 (9) (DG)	<LOD	[57]
	Bering Sea	2014	<LOD	1.4 (1), 1.6 (9) (DG)	<LOD	[57]
**Pen Shell (*Atrina vexillum*)**						
Mozambique	Inhaca Island	2020	NT	NT	7.7	[76]
***Tellina donacina***						
Spain	Galicia	2021–2022	<LOD	NT	63	[105]
**“Shellfish”**						
China	Beibu Gulf	2016	211	Low	NT	[33]
France		2005–2008	<LOD	90 total	<LOD	[15]
Greece			74	69 (1)	64	[39]
Italy		2002–2008	<LOD	105 total	<LOD	[15]
Netherlands	Ijmuiden	2007	<LOD	9.6 total	<LOD	[15]
Spain	Galicia	2017–2019	23.9	NT	NT	[37]

^a^ GYM A unless otherwise specified. DMD = 16-desmethyl GYM D. DG = digestive glands; ^b^ SPX analogues are labelled (1) to (9). (1) 13 desmethyl SPX C, (2) SPX A, (3) 13 desmethyl SPX D, (4) 13,19-didesmethyl SPX C, (5) SPX C, (6) isoSPX C, (7) 20-methyl SPX G, (8) SPX D, (9) 13,19-didesmethyl SPX C. DG = digestive glands; ^c^ PnTX G unless otherwise specified; NT = no data available; <LOD = below the limit of detection.

**Table 9:** Determination of a “safe” concentration of cyclic imines in seafood utilizing the oral mouse NOAEL and assuming a 70 kg person eats 400 g of shellfish.

	GYM	SPX	PnTX
NOAEL (µg/kg bodyweight)	7500	320	153
Apply safety factors (100×) (µg/kg bodyweight)	75	3.2	1.53
“Safe” concentration in seafood (µg/kg shellfish flesh)	13,125	560	268

See below for the origin of the NOAEL data.

## Data Availability

No new data were created or analysed in this study. Data sharing is not applicable to this article.
